# Using Al^3+^ to Tailor Graphene Oxide Nanochannels: Impact on Membrane Stability and Permeability

**DOI:** 10.3390/membranes12090871

**Published:** 2022-09-09

**Authors:** Yijing Y. Stehle, Ellen J. Robertson, Rebecca Cortez, Ivan V. Vlassiouk, Ronald B. Bucinell, Katelyn Olsson, Luke Kilby

**Affiliations:** 1 Department of Mechanical Engineering, Union College, Schenectady, NY 12308, USA; 2 Chemistry Department, Union College, Schenectady, NY 12308, USA; 3 Center for Nanophase Materials Sciences, Oak Ridge National Laboratory, Oak Ridge, TN 37831, USA

**Keywords:** graphene oxide, Al^3+^ modification, nanochannel, permeability, stability

## Abstract

Graphene oxide (GO) membranes, which form from the lamination of GO sheets, attract much attention due to their unique nanochannels. There is much interest in controlling the nanochannel structures and improving the aqueous stability of GO membranes so they can be effectively used in separation and filtration applications. This study employed a simple yet effective method of introducing trivalent aluminum cations to a GO sheet solution through the oxidation of aluminum foil, which modifies the nanochannels in the self-assembled GO membrane by increasing the inter-sheet distance while decreasing intra-sheet spacing. The Al^3+^ modification resulted in an increase in membrane stability in water, methanol, ethanol, and propanol, yet decreased membrane permeability to water and propanol. These changes were attributed to strong interactions between Al^3+^ and the membrane oxygenated functional groups, which resulted in an increase in membrane hydrophobicity and a decrease in the intra-sheet spacing as supported by surface tension, contact angle, atomic force microscopy, and X-ray photoelectron spectroscopy measurements. Our approach for forming Al^3+^ modified GO membranes provides a method for improving the aqueous stability and tailoring the permeation selectivity of GO membranes, which have the potential to be implemented in vapor separation and fuel purification applications.

## 1. Introduction

Graphene oxide (GO) sheets are monolayers of bonded sp^2^-hybridized carbon atoms decorated with abundant oxygenated functional groups (OFGs). These functional groups are located on the basal planes (hydroxyl, carbonyl, and carboxyl groups) and edges (carboxyl groups), as described by the widely accepted Lerf–Klinowski structural model [1,2,3]. GO membranes form from the lamination of individual GO sheets, which are prone to align parallel to a casting mold and assemble in a “brick-and-mortar” stacking configuration during drying on a solid surface [4,5]. During assembly, electrostatic and hydrogen bonding interactions between OFGs on individual adjacent GO sheets form nanochannels in GO membranes through which polar solvents and ions can permeate. These unique nanochannels in the laminated structure of GO membranes make them excellent candidates for separation and filtration applications [6,7,8,9,10,11,12,13]. To be useful materials in these applications, GO membranes must be tailored to be permeable to specific species of interest and exhibit good stability and structural integrity.

The presence of OFGs, however, can greatly affect both the permeability and aqueous stability of GO membranes. On the one hand, nanochannels within the GO membrane have the potential to be tunable and selective for specific chemical species based on the chemical modification to the OFGs. On the other hand, the hydrophilic OFGs can be hydrated by water; thus, the GO membranes readily dissolve in water. While strong π–π interactions exist between individual GO sheets in GO membranes as they do in graphene membranes, the introduction of OFGs weakens these interactions. Specifically, the OFGs cause the C-O sp^3^ carbons to become distorted, leading to amorphous regions of the membrane [1]. These structural defects weaken the strength and toughness of the laminated membranes, thus reducing their chemical, thermal, and aqueous stability. The instability of GO membranes in water prevents them from being used in aqueous environments, which is required in several filtration and separation applications [14,15,16,17,18,19,20,21]. Thus, strategies that could increase the stability of GO membranes while tailoring the selective permeability at the same time are of great interest. Previous studies demonstrated that either removing the OFGs or chemically cross-linking them to each other can increase the aqueous stability and control the permeability of GO membranes [3,6,19,20,21,22,23,24,25,26,27,28,29]. One of the most feasible and effective strategies to improve stability, however, is the crosslinking of OFGs on the GO sheets to multivalent metal cations [13,14,15,16]. The electrostatic and bonding interactions between multivalent cations and OFGs on the edges and basal planes of adjacent GO sheets can increase GO membrane stability by reducing negative surface charges and increasing adhesion between GO layers. Moreover, the ionic radii and valences of different metal cations can affect the interlayer spacing in GO membranes, which can in turn be used to tune the permeability of GO membranes for specific molecules and ions.

Previous studies revealed that trivalent cations such as Al^3+^ can interact with GO sheets through cation-π, coordination, and electrostatic interactions, which increases the interlayer spacing and improves both the integrity and water stability of the GO membrane [14,22]. Al^3+^ has benefits over other trivalent cations for stabilizing GO membranes, such as the ease and low cost of using aluminum foil as a source of the cation, as we previously reported [24]. Both theoretical and experimental studies on cation modified and unmodified GO membranes focused on the relationship between interlayer spacing, water flow, and stability of the GO membranes [20,21,22,23]. In these previous studies, however, the impact of intra-layer spacing on the nanochannel permeability to different molecular vapors was not explored, which could be critical for determining the types of environments in which these membranes can be used for filtering specific chemical species. Our previous study revealed that Al^3+^ cations can be added to the acidic GO solution with Al foil through chemical oxidation, which caused the solution pH to increase, the O/C ratio to decrease, and more wrinkles to form on the dried membrane surface [24]. In addition, the resultant Al^3+^-modified graphene oxide (AGO) membranes showed an increased inter-sheet distance, and a decreased intra-sheet spacing when compared with the unmodified GO membranes [24].

In this work, we explored how the structural changes to the GO membrane upon the addition of Al^3+^ affect the as-formed GO membrane stability in and its permeability to different polar solvents. Based on our previous results, this modification strategy induces crosslinking between the metal cations and the OFGs and decreases the density of OFGs, which we hypothesize will act to increase the overall stability of the membranes in polar solvents. Moreover, the decrease in the O/C ratio and the increase in the surface roughness as previously observed can increase the hydrophobicity of the GO membranes, which we hypothesize will specifically increase the aqueous stability of the membrane. Finally, we hypothesize that the increased inter-sheet distance and decreased intra-sheet spacing can allow for tailoring both the nanochannel structure and thus the membrane permeability to different solvents.

To test these hypotheses, we performed a series of experiments to characterize the properties of GO sheet solutions and membranes both with and without Al^3+^ modification. Specifically, we fabricated Al^3+^-modified graphene oxide (AGO) membranes with 0.3 wt.% Al, which is the highest addition that does not cause the membrane to crack, for scalable fabrication [24]. The AGO membranes were easily formed by adding Al foil to an acidic GO solution. In the acidic solution, the Al foil was oxidized, which gradually released Al^3+^ cations to the solution. Subsequently, individual GO sheets initially well-dispersed in an aqueous solution were brought together by crosslinking them to the positively charged Al^3+^ cations, followed by a slow self-assembly into an ordered laminated structure via drying the AGO solution on a Teflon evaporation dish. To determine the effect of Al^3+^ on the GO sheet solution, we compared the viscosity and surface tension of the AGO solution to the GO solution. The hydrophilicities and structures of the dried GO and AGO membranes were also compared using the contact angle, atomic force microscopy (AFM), and X-ray photoelectron spectroscopy (XPS) measurements. Finally, the stability of the GO and AGO membranes in different polar solvents as well as the vapor permeability of each membrane to these solvents were systematically assessed and explained through the inter- and intra-sheets structure. This study provides valuable guidance for developing GO membranes with tunable permeability and stability in different polar environments.

## 2. Materials and Methods

### 2.1. Materials and Membrane Fabrication

Graphene oxide water dispersions (4 mg/mL) were purchased from MSE Supplies, Tucson, AZ, USA. The aluminum (Al) foil (0.01 in. thick, Puratronic, 99.997% purity) with an area density of 0.65 mg/mm^2^ was purchased from Alfa Aesar, Haverhill, MA, USA. All reagents were used as received without further purification. Solutions of GO and Al (Al concentration = 0.3 wt.%) were prepared by dissolving the Al foil in a GO solution followed by continuous stirring for the complete dissolution of the Al foil and coordination between the GO sheets and the Al^3+^ cations. Free-standing GO and AGO membranes were obtained through a slow layer-by-layer self-assembly process in which the GO and AGO solutions were dried on glass slides. Ambient drying conditions were used to form all GO and AGO membranes to prevent membrane cracking and air bubble formation.

### 2.2. Characterization

The viscosities of the GO and AGO solutions were measured using a Brookfield DV-E Viscometer with an UL adapter spindle. Viscosity measurements were taken with a shear rate of 0.5~100 rpm. The surface tension measurements were performed using the pendant drop method on a Biolin Scientific Optical Tensiometer. Here, a hanging drop of the solution of interest was formed using an air-tight Hamilton syringe equipped with a blunt 22-gauge needle. Images of the droplet were collected approximately every minute for 30 min. The droplet shapes were fitted to the Laplace–Young equation using the OneAttension software provided by Biolin Scientific to determine the shape factor, *β*. The software then computed the air–fluid surface tension (*γ*, mN/m) using Equation (1), in which ∆*ρ* is the density difference between the air and solution, *R*_0_ is the radius of the droplet at the apex, and *g* is the acceleration due to gravity.

(1) $$\gamma =\frac{\Delta \rho R_{0}g}{\beta },$$

In all surface tension measurements, the droplets were encased in a quartz cuvette to prevent drop evaporation and agitation from air currents. The reported surface tension data are averages of at least 10 measurements. Contact angle measurements of dried GO and AGO membranes that had been drop-casted on glass microscope slides were performed using the Biolin Scientific Optical Tensiometer. In these measurements, data collection began immediately after a ~10 µL droplet of deionized water was dispensed onto a membrane of interest. Images of the droplet were taken over the course of 1 min at a rate of 0.68 frames per second. Contact angles were determined using the OneAttension software. The reported contact angle values were the averages of the right and left sides of the droplets. XPS data were acquired using a Thermo Scientific Model K-Alpha XPS instrument with a 400 µm X-ray spot size. Wide energy range survey spectra (0–1350 eV) were acquired by performing a subsequent analysis using an analyzer with a pass energy of 200 eV and energy step size of 1 eV.

### 2.3. Stability

To investigate the stability of the as-prepared GO and AGO membranes in different polar solvents, the dried membranes were cut into pieces with dimensions of about 0.6 cm × 0.6 cm and soaked in 15 mL of either ultrapure water, methanol, ethanol, or propanol at room temperature. Four different stability studies were performed that varied the extent to which the membranes were agitated in the solvents. In the first study, the membranes were soaked in the solvents with no agitation. In the second study, the membranes were gently stirred using a 10 mm long × 3 mm diameter stir bar that rotated at a rate of 200 rpm. In these studies, which were performed in 20 mL scintillation vials, the stir bar was small enough that it did not collide with the membranes during stirring. In the third study, the membranes were vigorously stirred using a 12 mm long × 5 mm diameter stir bar that rotated at a rate of 200 rpm. In the 20 mL scintillation vials, the larger stir bar continuously collided with the membrane during stirring. In the fourth study, the membranes were sonicated using a 60 Hz sonicator. Images of the membranes were collected over time for each stability study using a digital camera to assess the extent of membrane dissolution.

### 2.4. Permeability

The permeability of GO and AGO membranes to different solvents was assessed using a simple evaporation device as shown in Figure S1 (in Supplementary Materials). The free-standing GO and AGO membranes were cut into circular shapes with diameters of 2 cm and sealed on the top of a glass vial. The setup was placed in an atmospheric pressure chamber with a constant temperature and humidity. The effective permeation area (A) for the setup was 1.27 cm^2^. Ultrapure water and high purity methanol, ethanol, and propanol were used for permeability tests at temperatures of 30, 40, 50, 60, and 70 °C. The weight difference (*M*, g) of the solvent was measured by periodically monitoring the mass of the vial for up to seven days and used in the permeation rate calculation. The flux (*J*, g m^−2^ hr^−1^) of solvent molecules across membranes was obtained by finding the slope of the curve between the amount of solute molecule permeated per unit membrane area (n/A) and the permeation time (*t*), according to Equation (2). Each recorded permeability value is the mean of at least 5 experiments at the same temperature. According to Fick’s first law (Equation (3)), the flux through a thin membrane is proportional to the diffusion coefficient (*D*, m^2^/h), concentration of vapor on the two sides of the membrane (Δ*C*), and the reciprocal of the membrane thickness (Δ*x*).

(2) $$J=\frac{M}{A•t}$$

(3) $$J=\frac{-D\Delta C}{\Delta x}$$

## 3. Results and Discussion

**Figure 1:**
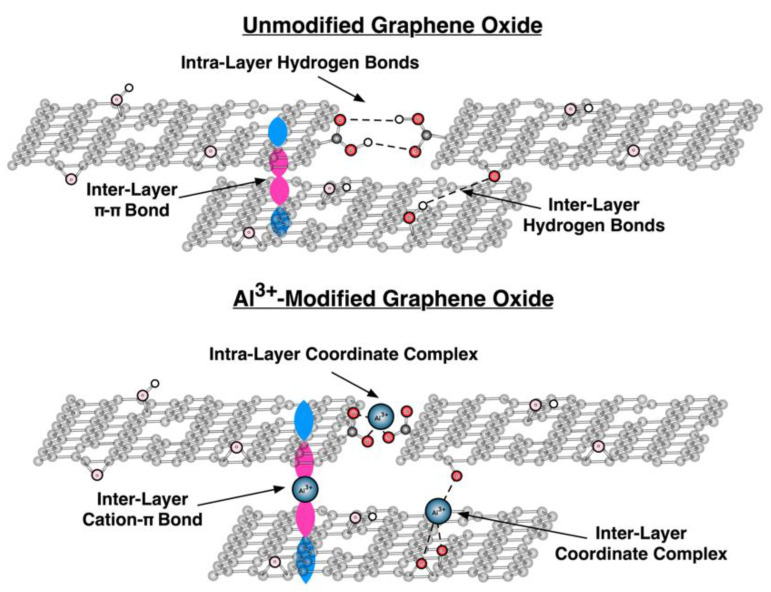
Schematic of the nanochannel in both unmodified (**top panel**) and Al^3+^-modified (**bottom panel**) GO membranes. The addition of Al^3+^ to the GO membrane results in an increase in the inter-layer distance and a decrease in the intra-layer spacing.

It was previously reported that pristine GO membranes are completely impermeable to all gasses and liquids except water vapor due to their unique nanochannel structure [3]. The major inner-membrane structures that dominate the nanochannels are inter-layer galleries, intra-layer frameworks, and vacancy defects [9,12]. These nanochannels are more generally spaces in the membrane through which molecules can permeate, rather than well-defined tubes that can be experimentally visualized and quantified. The inter-layer and intra-layers structures play an important role in the stacking of the GO nanosheets within the membrane. This stacking affects the nanochannel size, which in turn affects the membrane stability and permeability. The enlarging of the GO nanochannel by increasing either interlayer or intra-layer spacing can accelerate the water flow or permit larger vapor molecular to pass through the membrane. It was previously reported that for membranes modified with cations of the same valency, a larger interlayer spacing results in greater water permeation if the intra-layer spacing change is negligible [19]. Multivalent metal cation modification is effective at both improving the aqueous stability of the GO membrane and increasing the interplanar distance, but this modification does not always result in increased water permeation [15,19]. Because it is difficult to experimentally measure the intra-sheet spacing, previous studies focused on understanding the effects of the interplanar (inter-layer) distance on the nanochannel structure and the membrane stability [9,20,21,22,23]. In our previous study and as demonstrated in Figure 1, we determined that modification of the GO membrane with Al^3+^ via oxidation of Al foil resulted in an increase in the membrane inter-layer distance, which was previously reported, and in a decrease in the membrane intra-layer spacing, which is not often discussed [24]. In the studies described below, we related the changes in the inter- and intra-layer distances upon Al^3+^ modification of the GO membrane to the membrane properties as well as stability in and permeability to different polar solvents.

### 3.1. Solution and Membrane Characterization

The GO solution in the absence of Al^3+^ is a liquid crystal. Its rheological-related properties such as viscosity determine the applicable fabrication methods, which may include electrospraying, wet/dry spinning, inkjet printing techniques, etc. [30,31,32,33]. Unlike the unmodified GO solution, the AGO solution exhibited a “muddy” appearance (Figure S2), which was initially considered to be due to the formation of a gel. To fully understand the “muddy” appearance of the AGO solution, the viscosity of the unmodified GO and AGO solutions (Figure S3) was measured. Both the GO and AGO solutions exhibited a typical and similar pseudoplastic behavior, or so-called viscoelastic behavior, which means the viscosity (η) decreases and the shear stress (τ) increases with the shearing rate. Thus, the solution viscosity cannot explain the “muddy” appearance of the AGO solution. Instead, it is likely that the change in consistency of the sheet solution upon the addition of the Al foil was due to interactions of the Al^3+^ cations in solution with the OFGs on the GO sheet surfaces.

**Figure 2:**
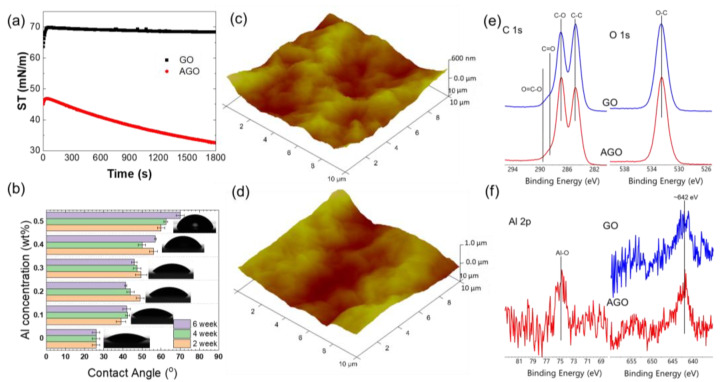
Effect of Al^3+^ modification on GO and AGO sheet solutions and membranes formed via the layer-by-layer self-assembly method. The influence of Al^3+^ on the (**a**) air–water interfacial surface tension of a GO sheet solution, (**b**) contact angle of GO membranes assembled on glass slides; AFM image of (**c**) pristine GO (height scale bar 600 nm) and (**d**) AGO (height scale bar 1 µm) membranes on thermal silicon oxide wafers; XPS spectra of (**e**) C 1s and (**f**) O 1s spectra of GO and AGO membranes.

The interactions between Al^3+^ and the GO sheets is supported by surface tension measurements of the GO and AGO solutions. These surface tension measurements provide information about the extent of adsorption of a species in solution to the air–water interface. If the surface tension of a solution does not decrease much relative to that of the neat air–water interface (72 mN/m), then the species in the solution is not highly surface active. If the surface tension of a solution significantly decreases relative to that of the neat air–water interface, then the species is surface active. The average surface tension vs. time data were collected for both GO and AGO solutions using a pendant drop tensiometer and are summarized in Figure 2a. In the absence of Al^3+^, the surface tension of the GO solution initially increased from approximately 60 mN/m to approximately 70 mN/m over the course of around one minute. After this rapid increase, the surface tension did not significantly change over time. The fact that the surface tension is similar to the neat air–water interface suggests that GO sheets are not highly surface active and likely desorbed from the interface over the first minute of interface formation. This result is consistent with the surface structure of GO, in which the negatively charged OFGs cause the material to be highly water-soluble. In the presence of Al^3+^, the surface tension of the AGO solution showed a small initial increase followed by a decrease to approximately 30 mN/m over the course of 1800 s. The decrease in surface tension over time indicates that AGO sheets continually adsorb species to the air–water interface, potentially forming multiple layers through OFG-Al^3+^ interactions. The surface tension data suggest that the addition of Al^3+^ can impact the self-assembling process of the GO sheets at the air–water interface, which acts to increase the hydrophobicity of the sheets and decreases their solubility in water.

Our previous report showed that releasing Al^3+^ in the GO solution via the oxidation of Al foil is a slow process and results in an increase in the solution pH [24]. The protonation of the OFGs is highly sensitive to the solution pH, which in turn affects the extent of crosslinking between the OFGs and Al^3+^. Thus, it is likely that the hydrophobicity of the AGO membrane is impacted by both the Al^3+^ concentration and the time at which the GO solution is exposed to the Al foil before membrane formation. The relative hydrophobicities of GO membranes formed from solutions that were exposed to Al foil for varying times were determined from contact angle measurements, as presented in Figure 2b. Here, the contact angles of ultrapure water droplets with membranes casted on the glass slide surfaces were used to estimate the hydrophobicity of the GO and AGO membranes. As shown in Figure 2b, the contact angle of the GO membrane increased with the Al^3+^ concentration, which indicates an increase in the hydrophobicity of AGO membranes when compared to the GO membranes. The increase in sheet hydrophobicity, as observed in the surface tension and contact angle measurements, is consistent with the interaction of the positively charged Al^3+^ cations with the negatively charged OFGs on the sheet surfaces, either through electrostatic screening or chelation. Additionally, the increase in the density of wrinkles formed with the addition of Al^3+^ can further increase the hydrophobicity of the GO membranes [24].

The interactions between Al^3+^ and the OFGs not only affect membrane hydrophobicity, but also the presence of membrane features such as wrinkles, shrinkage, and cracks. Specifically, it was observed that the muddy-like AGO solutions with an Al wt.% of greater than 0.3 wt.% formed membranes that, upon drying, demonstrated a significant decrease in diameter and excessive cracking. Significant membrane shrinking and cracking was not observed for membranes formed from less muddy-like AGO solutions with an Al wt.% of less than 0.3 wt.% (Figure S4). The decrease in the membrane diameter with an increase in the Al wt.% supports the previous conclusion that the addition of Al^3+^ decreases the intra-sheet spacing [24].

The “brick-and-mortar” stacking configuration of laminated GO sheets introduces mixed interactions among the basal planes and edges, which lead to ‘peak and valley’ undulations in the membrane structure. The accumulation of these undulations results in wrinkles, which increases the surface roughness of the membrane [1]. Our previous report shows that Al^3+^ modification via Al foil oxidation causes more wrinkles to form on both GO sheets and membranes, which can lead to an increase in surface roughness [24]. Stronger interactions between Al^3+^ and the OFGs on GO sheet surfaces can increase the ‘peak and valley’ undulations and thus cause more wrinkles in the AGO membrane compared to the GO membrane. Three-dimensional AFM images of the GO (Figure 2c) and AGO (Figure 2d) membranes can be used to estimate the average surface roughness (RA) of as-fabricated GO and AGO membranes. According to the average RA value collected from five AFM images of the GO and AGO membranes, the AGO membrane had an RA roughness value of 98nm, while the value of pure GO membrane was 87nm, which is consistent with the greater density of wrinkles in the AGO membrane when compared to the GO membrane [24].

To better understand the effect of Al^3+^ on the chemical composition of the dried GO membranes, XPS data were collected and are shown in Figure 2e,f. XPS is a surface-sensitive technique that can be used to effectively determine the elemental composition and chemical environment of a GO membrane within the first few nanometers of its surface. Figure 2e, which provides the overall surface composition of the GO and AGO membranes, demonstrates that carbon and oxygen comprised more than 98 at. % of the surface. Moreover, the C/O ratio varied between 2.3 and 2.6 for all GO and AGO membranes studied. These results are consistent with the presence of hydroxyl, epoxy, and carboxyl groups on the membrane surfaces, as expected. All membranes showed peaks due to small amounts of N and S, while only the AGO membranes showed a small peak due to the presence of Al near 75 eV (Figure 2e). As shown in Figure 2f, the primary C 1s peaks at 284.5 eV and 286.8 eV confirm the presence of C–C (or C−C/C=C) and C-O (C−O/C−N, C−O−C) functional groups, respectively. Figure 2f also shows two small C 1s peaks at 288.8 eV and 289.4 eV, which indicate the presence of O-C=O and C=O functional groups. The primary O 1s peak at 532.3 eV is attributed to the presence of OFGs within the GO membrane. Importantly, the introduction of Al^3+^ to the GO membrane resulted in a decrease in the C-C peak area and an increase in the C-O peak area in the C 1s spectra. This result can be attributed to the coordination between Al^3+^ and epoxide groups that underwent ring opening reactions.

### 3.2. Membrane Stability

The instability of GO membranes in aqueous environments is a long-standing problem for applications that require GO membranes to be used in polar solvents, which is due to the chemical structure of the GO sheets that compose the membranes [10,11,12]. Specifically, the hydrophilic OFGs on GO sheets cause both the sheets and laminated membranes to become hydrophilic and hold a negative surface potential. The electrostatic repulsions between negatively charged GO sheets within the membranes can overcome the van der Waals attractions and hydrogen bonding interactions between sheets in the aqueous environment, especially in the presence of shear force and sonication energy [12]. The low contact angle of unmodified GO indicates the high tendency of GO sheets to be solvated by water molecules after becoming fully soaked, which further decreases the extent of interactions between adjacent GO sheets within the GO membrane.

**Figure 3:**
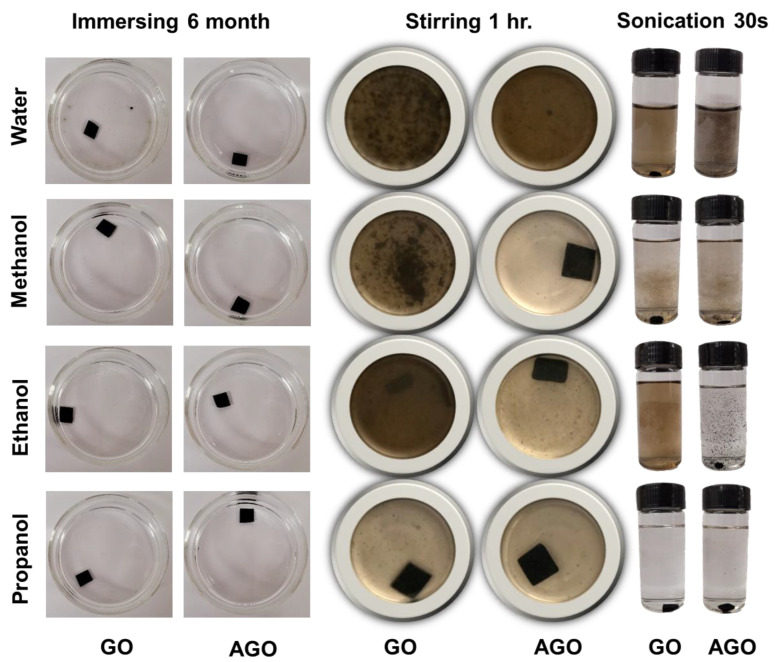
Stability of the GO and AGO membranes in water, methanol, ethanol, and propanol. The GO and AGO membranes were soaked (**left**), vigorously stirred (**middle**), and sonicated (**right**) in different polar solvents for different time intervals.

In this study, the stabilities of GO and AGO membranes in water and three different polar solvents were assessed under three different degrees of agitation including soaking, stirring, and sonication. The results of this study are illustrated in the digital camera photos shown in Figure 3 and Figures S5–S8. Compared to GO membranes typically prepared through the vacuum filtration process, GO membranes prepared with layer-by-layer self-assembly demonstrate better stability due to the greater number of hydrogen bonds formed between individual GO sheets during the slow-drying process [2,3]. Specifically, previous studies demonstrated that GO membranes formed via vacuum filtration are only stable in water for up to an hour after soaking [2]. The results here show that the as-fabricated unmodified GO membranes are stable and maintain their original morphologies after being soaked in pure water as well as methanol, ethanol, and propanol for more than 6 months (Figure 3, column 1; Figure S5) [24].

However, agitation of the membranes decreased their stability over time. Under a sonication frequency of 60 Hz, the GO membranes in water, methanol, and ethanol disintegrated within 30 s of sonication(Figure 3, column 5), while the GO membranes in propanol maintained their integrity during 60 s of sonication (Figure S6). Agitation of the membranes via stirring (Figure 3, column 3), which is more representative of a filtration working environment, resulted in GO membranes that were less stable than those soaked in the solvents with no agitation, but more stable than those subjected to sonication. This result is consistent with the fact that the shear force generated by stirring is much lower than the shear force generated through sonication. During stirring, the disintegration of the GO membrane in water exhibited an edge-to-center (intra-layer) disassembly pattern rather than a layer-by-layer (inter-layer) disassembly pattern when the time of stirring increased. The complete dispersion of the unmodified GO membrane in water required about 96 h of stirring. The rate of membrane agitation, and thus rate of disintegration, was adjusted by increasing the size of the stir bar. Because the solvent volume and sample vial used remained the same, the increase in the stir bar size resulted in collisions between the stir bar and membrane during each stir bar rotation. For GO membranes in water, membrane disintegration became visible after 20 min of stirring with a larger stir bar, with complete dispersion occurring within 1 h (Figure S7). Here, the rate of membrane disintegration was much faster than for membranes agitated by a smaller stir bar because the membrane was exposed to both the force of the shear stress and the impact of the stir bar (Figures S7 and S8).

As seen in Figure 3, the stability of the unmodified GO membrane decreased with decreasing solvent hydrophobicity, such that the membrane was most stable in propanol and least stable in water. The instability of the GO membrane in aqueous solution results from the hydration of OFGs that are both exposed on the surface of the membrane and embedded in the membrane interior [34]. Specifically, hydrogen bonding, ion-dipole, and dipole–dipole interactions between water molecules and hydrophilic OFGs on the surface of the GO membrane can overcome the relatively weak van der Waals and hydrogen bonding interactions that hold the surface-exposed sheets together. Additionally, the size of a water molecule is likely smaller than the inter-sheet lamellar distance, or intra-sheet framework space, and so it can permeate into the membrane interior. Here, interactions between water molecules and OFGs located in the membrane interior can increase the inter-sheet distances, thereby weakening the π-π interactions that hold individual sheets together in the membrane. The increase in membrane stability with increasing solvent hydrophobicity is likely due to both a decrease in the extent of interactions between the solvent alcohol groups and surface-exposed membrane OFGs and an inability to permeate into the membrane interior due to the increasing molecular size beyond that of the critical lamellar distance.

Previous work demonstrated that the water stability AGO membranes formed via pre-coordination followed by vacuum filtration is only improved under soaking conditions for up to a few hours [14,22]. In our study, AGO membranes that were formed via pre-coordination followed by a slow layer-by-layer self-assembly process remained stable under soaking conditions in water, methanol, ethanol, and propanol for more than 6 months (Figure S5). Thus, to assess the effect of Al^3+^ modification on GO membrane stability, the responses of the as-prepared GO and AGO membranes to stirring and sonication in the different polar solvents were compared (Figure 3 and Figures S6–S8). These results show that when the membranes are agitated by both stirring and sonication, the AGO membranes show a better stability and survive for longer than the unmodified GO membranes in all four different polar solvents.

However, consistent with previous studies of GO membranes formed with vacuum filtration, improvements in membrane stability were not dramatic when GO membranes were modified with Al^3+^ via the pre-coordination method [2]. As seen in Figures S6–S8, the AGO membranes still disintegrated in water, methanol, and ethanol upon increasing the time of stirring and sonication. Both GO and AGO membranes showed good stability in propanol and did not disintegrate after 60 s of sonication. Moreover, the process by which the GO and AGO membranes dissolved in pure water under stirring and sonication were observed to be quite different. In water, the GO membrane disintegrated uniformly into a well-dispersed brown solution with no visible particles or flakes, as observed by the eye. Under the same conditions as the GO membrane, the AGO membrane disintegrated non-uniformly into large black flakes consisting of AGO sheet aggregates that could be seen by eye. In the water solution, the disintegrated AGO membrane existed as large black colored flakes. After sonication and storage, these flakes persisted in water, methanol, and ethanol (Figure S6). The persistence of the AGO flakes compared to the uniformly dispersed GO membranes further demonstrates that Al^3+^ modification increases the stability of GO membranes in polar solvents. This increased membrane stability was achieved by replacing inter-sheet hydrogen bonds with cation-to-OFGs coordinate covalent bonds, cation-π interactions, and electrostatic interactions. In addition to decreasing membrane hydrophilicity by screening the negative charges on the membrane surface, these interactions are overall stronger than inter-sheet hydrogen bonds. Both factors are critical for increasing the stability of GO membranes in polar solvents.

It is likely that the limited stability improvement of the AGO membrane is in part due to its reduced water permeability. The AGO membranes modified with Al^3+^ from Al foil exhibited increasing membrane wrinkling, shrinking, and cracking after increasing the Al wt.% in the GO solution (Figure S4). The shrinking of the AGO membrane supports our previous report [24], which showed a decrease in the intra-sheet spacing after increasing the Al^3+^ wt.%. This membrane diameter decrease is consistent with stronger intra-layer interactions and increased membrane stability. However, the highly wrinkled and contracted AGO membrane exhibited swelling after being fully soaked in water, suggesting that the membrane is permeable to water (Figure S9). The fact that water can intercalate into the membrane interior likely prevents the membrane from being completely stable in water under all agitation conditions. The extent of AGO membrane swelling compared to the extent of GO membrane swelling in ultrapure water could not be reliably quantified (Figure S10); therefore, it was difficult to assess the effect of Al^3+^ modification on membrane permeability purely from soaking in water.

In summary, the unmodified GO membrane prepared using the slow layer-by-layer self-assembling process can maintain its integrity when soaked in water, methanol, ethanol, and propanol, but will severely disintegrate and disperse into water, methanol, or ethanol within 30 s of vigorous sonication or 2 h of stirring. The stability of GO in methanol and ethanol is better than that in water, while the best stability is observed in propanol. Overall, AGO membranes demonstrated enhanced stability in water, methanol, and ethanol, but this improvement was limited.

### 3.3. Membrane Permeability

**Figure 4:**
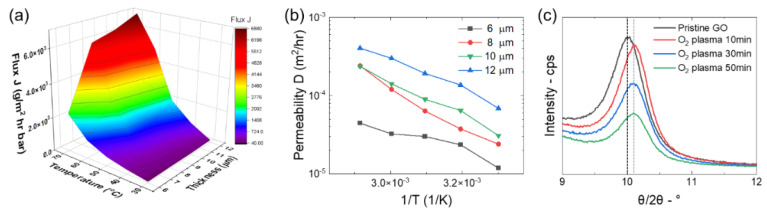
Permeability of a GO membrane to water as a function of membrane thickness and temperature. (**a**) Flux of water vapor through GO membranes with thicknesses varying from 8~12 µm and temperatures ranging from 30 to70 °C. (**b**) Corresponding permeability values derived from (**a**). (**c**) XRD spectra of GO membrane before and after exposure to oxygen plasma.

In this study, the membrane permeability of both GO and AGO to water, methanol, ethanol, and propanol vapor was assessed by measuring the loss of solvent mass from a bottle that was capped with the membrane of interest (Figure S1). From the loss of mass, the flux, *J*, was calculated using Equation (2), and subsequently the diffusion coefficient, *D*, was derived using Equation (3), which was equated to the membrane permeability. The flux of water vapor passing through membranes with thicknesses ranging from 6 µM to 12 µM and at temperatures ranging from 30 to 70 °C was calculated first (Figure 4a). We were unable to measure the permeability of thinner membranes due to their fragility and thicker membranes due to their extensive wrinkling and inability to properly seal the permeability set-up. It is expected that as the temperature increases, the flux of water vapor through the membrane will increase due to an increase in the kinetic energy, and thus speed, of the vapor molecules passing through the membrane. Moreover, as membrane thickness increases, it is expected from Fick’s Law that the flux of water vapor through the membrane will decrease due to an increase in the amount of time the molecules can spend in the thicker membrane. As expected, the flux of water vapor through the GO membrane increased with an increasing temperature; however, the flux also increased with increasing membrane thickness. The flux data were used to calculate the permeability, *D*, for membranes at each thickness and temperature (Figure 4b). These data indicate that the permeability also increased with increasing temperatures and membrane thickness.

The increase in permeability with increasing membrane thickness was unexpected, and likely resulted from how the individual GO sheets were structured differently at the surface of the membrane compared to the interior of the membrane during drying in the layer-by-layer self-assembly process. As suggested in the surface tension data and observed during the experiment, GO sheets accumulated at the air–water interface and dried first from the surface to the interior during the self-assembly process. To explore the effect of membrane thickness on the inter-sheet structure at the surface of the GO membrane, XRD spectra of GO membranes were collected with varying thicknesses (Figure S11). These results reveal no obvious trend in the average interplanar distance of GO membranes with different thicknesses. Thus, it is likely that the surface structure of the membrane does not depend on the membrane thickness. However, it is possible that the membrane surface structure is significantly different than the structure of the membrane interior, which could affect the flux of water through membranes with different thicknesses.

To determine the difference of the inter-sheet structure between the GO membrane surface and GO membrane interior, XRD spectra of membranes were collected before and after exposure to oxygen plasma for varying times (Figure 4c). Here, a radio-frequency oxygen plasma at the processing power of 60 W from 10 to 60 min was applied to remove the surface of the GO membrane before each XRD scan. The resultant spectra show a slight shift of the peak from 10.01°, corresponding to an average interlayer distance of 8.826 nm, to 10.11°, corresponding to an average interlayer distance of 8.739 nm. The decrease in the average inter-sheet distance after removal of the surface suggests that the sheets near the membrane surface are somewhat more densely packed in the vertical direction than sheets in the membrane interior. The formation of the densely packed membrane interior occurs during the membrane drying process. It is likely that the GO sheets that assemble at the air–water interface form a permeation layer that inhibits the evaporation of water molecules from the bulk solution and slows down the drying process. This can result in a uniformly assembled membrane interior structure through which water molecules have to slowly permeate before reaching the surface.

It is expected that as the membrane thickness increases and the interior structure rather than the surface structure dominates the properties of the membrane, permeability decreases due to the decrease in the inter-sheet distance in the membrane interior. However, the permeation channel is controlled by the inter-sheet distance, the intra-sheet spacing, and vacancy defects on the GO sheet. Specifically, and as we show in our AGO studies below, an increase in the inter-sheet distance does not increase the membrane permeability due to the decreased intra-sheet spacing. The finding that the permeation coefficient increases with membrane thickness and larger inter-sheet distances between sheets near the surface than in the interior supports our hypothesis that the inter-sheet distance does not dominate the permeation channel of the GO membrane. However, the intra-sheet structure difference between the surface and the interior of the membrane is unclear. Moreover, it is also unclear if the ambient oxidation of the surface during the week-long drying process occurs, which could also affect the nanochannel structure.

**Figure 5:**
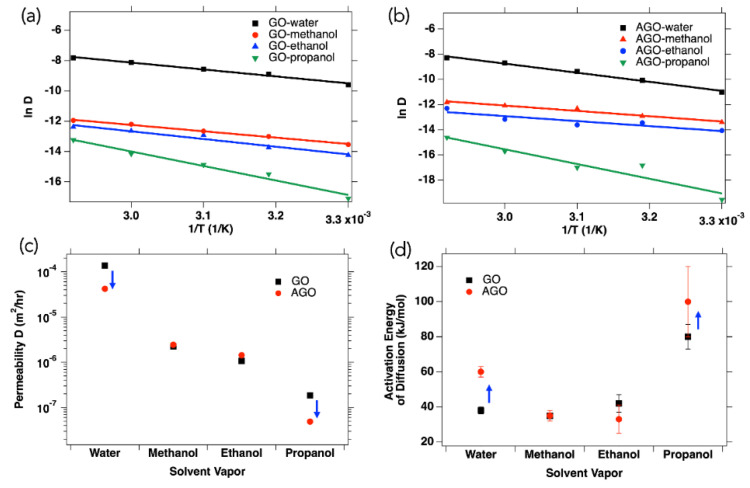
Arrhenius plots for (**a**) GO permeability and (**b**) AGO permeability to water, methanol, ethanol, and propanol. The lines in (**a**,**b**) are fit to Equation (4). (**c**) Permeability of GO and AGO membranes to different polar solvents at 30 °C. (**d**) Activation energies of diffusion for water, methanol, ethanol, and propanol through the GO and AGO membranes.

Previous reports revealed that metal cation-decorated GO membranes exhibited selective permeation to organic solvents [27], which is consistent with the results presented here. To determine the effect of Al^3+^ modification on membrane permeability, the permeabilities of 12 μm thick GO and AGO membranes to four different polar solvents at temperatures ranging from 30 °C to 70 °C were compared (Figure 5). A summary of the temperature-dependent data is provided in Figure S12. Here, the temperature dependent permeability data for the GO membrane (Figure 5a) and the AGO membrane (Figure 5b) were plotted according to the Arrhenius equation (Equation (4)) to better understand the kinetic parameters affecting GO and AGO membrane permeability to different polar solvents.

(4) $$lnD=lnD{}^{\circ}-\frac{Q_{d}}{RT}$$

Here, *D°* is the pre-exponential factor, *Q_d_* is the energy of activation of diffusion, *T* is the absolute temperature in Kelvin, and *R* (8.314 J/mol K) is the gas constant. As expected, the permeability of both GO and AGO to all solvents increased with increased temperature. The GO membrane has a water vapor flux of 68 ± 5 g/m^2^h at 30 °C, which is consistent with other reports [11]. It is impressive that the flux of water vapor across the 12 µm thick GO membranes can go up to 600 g/m^2^h at 70 °C, which is almost the same as the vaporization rate of water at the same temperature.

At all temperatures studied, both membranes showed an increase in permeability with a decreasing molecular size, such that the 12 µm thick GO and AGO membranes were most permeable to water and least permeable to propanol. Without the effect of hydration by water vapor present in the atmosphere, the GO membrane permeabilities to methanol, ethanol, and propanol (Figure 5c) were 1.32 × 10^−6^, 6.57 × 10^−7^, and 3.76 × 10^−8^ m^2^/h, respectively, at 30 °C, which were much lower that the water vapor permeability at the same temperature (6.85 × 10^−6^ m^2^/h). This result is consistent with the stability studies, which showed that membranes were most stable in propanol and least stable in water under vigorous stirring and sonication. A similar explanation to that of the the stability data can be used for the permeability data. Specifically, the GO membrane was most permeable to water because it is likely that the nanochannels are larger than the size of a water molecule. As the molecular size increases relative to the size of the nanochannels, the vapor becomes less permeable to the membrane. Another possible effect that was previously reported is the effect of water vapor present in the atmosphere on membrane permeability [11,27]. In this case, intercalating water molecules in GO-based membranes either block or impede the alcohol molecules from passing through the membranes. As seen in Figure 5c, Al^3+^ modification decreased membrane permeability to both water and propanol vapor, while having little effect on permeability to methanol and ethanol vapor. At 30 °C, the permeability to water decreased to 1.65 × 10^−6^ m^2^/h (76% decrease) and the permeability to propanol decreased to 3.20 × 10^−9^ m^2^/h (91% decrease).

To see if the change in membrane permeability to different solvents was related to the activation energy of diffusion, the lnD vs. 1/T data in Figure 5a,b were fit to Equation (4). The activation energies, which can be used to quantify the energy barrier, that the vapor molecule must overcome to diffuse through the GO or AGO membrane are reported in Figure 5d. From these results, it can be seen that upon Al^3+^ modification of the GO membrane, the activation energy of water vapor diffusion increased from 38 ± 2 kJ/mol to 60 ± 3 kJ/mol, the activation energy of propanol diffusion increased from 80 ± 7 kJ/mol to 100 ± 20 kJ/mol, and the activation energies for methanol and ethanol diffusion did not significantly change. Thus, it was possible to relate a decrease in membrane permeability to an increase in the activation energy of diffusion for a given solvent through the membrane.

The decrease in GO membrane permeability to water, and the corresponding increase in the activation energy of diffusion, upon Al^3+^ modification, is consistent with our previous studies of GO and AGO membranes formed via the layer-by-layer self-assembly method [24]. Our studies demonstrated that modification via post-cation intercalation leads to GO membranes that possess controllable thicknesses and interlayer spacings. Specifically, it was discovered that crosslinking Al^3+^ to the OFGs in the GO membrane resulted in an increase in the inter-layer distance and a decrease in intra-layer spacing. The intra-layer framework of the nanochannels impacted the water vapor flow more than the inter-layer gallery, which resulted in both longer and narrower nanochannels than those in unmodified membranes as shown in Figure 1. Moreover, the surface tension and contact angle data presented here indicate that Al^3+^ modification increases the hydrophobicity of the GO membrane due to interactions between the cations and the negatively charged OFGs. Both a decrease in intra-layer spacing and an increase in membrane hydrophobicity can decrease membrane permeability to water vapor. In the first case, the narrowing of the nanochannels due to Al^3+^ crosslinking can block water molecules from permeating through the membrane. In the second case, the edge-to-edge coordination of Al^3+^ with the carboxylate groups on the edges of the GO sheets can inhibit permeability by consuming the OFGs of GO sheets such that water molecules cannot interact with these hydrophilic groups. For methanol, ethanol, and propanol, which are all more hydrophobic than water, it is likely that the increase in membrane hydrophobicity upon Al^3+^ modification does not greatly affect GO membrane permeability to these solvent vapors. For propanol, however, it is likely that the decrease in intra-layer spacing greatly reduces the GO membrane permeability to this relatively large vapor molecule, whereas no significant effect is observed in the GO membrane permeability to the smaller methanol and ethanol vapor molecules upon Al^3+^ modification.

## 4. Conclusions

In summary, Al^3+^-modified GO membranes were formed by directly dissolving Al foil into an acidic GO sheet solution. The coordination interactions formed between Al^3+^ and the OFGs on individual GO sheets in solution impacted the assembly process and resulted in an inter-layer distance increases and an intra-layer spacing decreases. The addition of Al^3+^ affected the properties of both the GO solution and as-formed membrane. When added to the GO sheet solution, the Al^3+^ caused the solution to appear “muddy” and resulted in a decrease in the solution surface tension. After drying during the layer-by-layer self-assembly process, AGO membranes showed a higher surface roughness, were more hydrophobic, and exhibited shrinkage with more cracking than unmodified GO membranes. Studies of GO membranes under soaking, stirring, and sonication in different polar solvents revealed an increase in membrane stability with increasing solvent hydrophobicity such that membranes were most stable in propanol and least stable in pure water. Modification of the GO membrane with Al^3+^ resulted in AGO membranes with improved stability in aqueous environments. This increased stability is likely the result of both a decrease in membrane hydrophilicity and an increase in inter and intra-layer interactions due to the coordination between Al^3+^ and the negatively charged OFGs on the GO sheet edges and basal planes. The permeability of the GO and AGO membranes to water, methanol, ethanol, and propanol vapor was explored using a simple apparatus. The results showed that both GO and AGO membranes were more permeable to smaller solvent molecules. Moreover, the permeability of the GO membrane to water and propanol decreased with Al^3+^ modification, with no effects observed in permeability to methanol or ethanol. The decrease in permeability was related to an increase in the activation energy of diffusion. The decrease in membrane permeability to different polar vapors with Al^3+^ modification was likely due to both a decrease in nanochannel size and an increase in membrane hydrophobicity that resulted from crosslinking between Al^3+^ and the OFGs on the edges of the GO sheets within the membrane. Although an increase in permeability might be expected with an increase in inter-sheet distance, the decrease in intra-sheet spacing appeared to dominate the GO membrane nanochannel structure and permeability. A complete understanding of how Al^3+^ modification impacts the GO membrane self-assembling process, its nanochannel structure, properties, and performance is still limited.Our approach provides a simple and cost-effective strategy to tailor the stability and permeation selectivity of GO membranes for potential water treatment and fuel dehydration applications by using Al^3+^ to specifically tune the inner structure of the GO membrane.

## Data Availability

Not applicable.

## Supplementary Materials

The following supporting information can be downloaded at: https://www.mdpi.com/article/10.3390/membranes12090871/s1, Figure S1: Schematic (**a**) and camera picture (**b**) of the permeability experiment set up; Figure S2: The AGO solution has a “muddy” look when compared with the GO solution; Figure S3: Viscosity and shear stress at different shear rates of GO solutions with Al concentrations from 0~0.5 wt.%; Figure S4: Digital camera image of as-fabricated GO and AGO membrane in the evaporation dish of the same size; Figure S5: Soaking stability test of GO/AGO membranes in solution with different solvents; Figure S6: Sonication stability test of GO/AGO membranes in solution with different solvents; Figure S7: Stability test of GO and AGO membranes in different solvents with a stirring rate of 200 Rpm with a large stir bar (12 mm Length × 5 mm Diameter) touches the sample during stirring; Figure S8: Stability test of GO and AGO membranes in different solvents with a stirring rate of 200 Rpm with a small stir bar (10 mm Length × 3 mm Diameter) bar not touch the sample during stirring; Figure S9: A highly wrinkled and contracted AGO membrane (**left**) swelled after soaking in water for 48 h(right); Figure S10: GO (**left column**) and AGO (**middle** and **right columns**) membrane soaking. The swelling cannot be visibly observed for the GO membrane and AGO membrane with Al wt.% < 1wt.%; Figure S11: (**a**) XRD spectra of GO membrane with different thicknesses, and (**b**) distribution of interplanar distance derived from XRD spectra; Figure S12: Permeability of GO and AGO membranes to water, methanol, ethanol, and propanol water at different temperatures.
