# Supplementary material for: Using Al3+ to Tailor Graphene Oxide Nanochannels: Impact on Membrane Stability and Permeability
Source: Membranes (Basel). 2022 Sep 9;12(9):871. doi: 10.3390/membranes12090871 (PMC9502523; doi:10.3390/membranes12090871)
Supplement: Supplementary file 1 [file membranes-12-00871-s001.zip › membranes-1907171-supplementary.pdf]

Supplementary Information

Using Al^{3+} to Tailor Graphene Oxide Nanochannels: Impact on Membrane Stability and Permeability

Yijing Y. Stehle[1,*], Ellen Jean Robertson[2,*], Rebecca Cortez[1], Ivan V. Vlassiouk[3], Ronald B. Bucinell[1], Katelyn Olsson[1], and Luke Kilby[1]

[1] Department of Mechanical Engineering, Union College, Schenectady, NY 12308, USA
[2] Chemistry Department, Union College, Schenectady, NY 12308, USA
[3] Center for Nanophase Materials Sciences, Oak Ridge National Laboratory, Oak Ridge, TN, 37831, USA
* Correspondence: stehley@union.edu; roberte2@union.edu

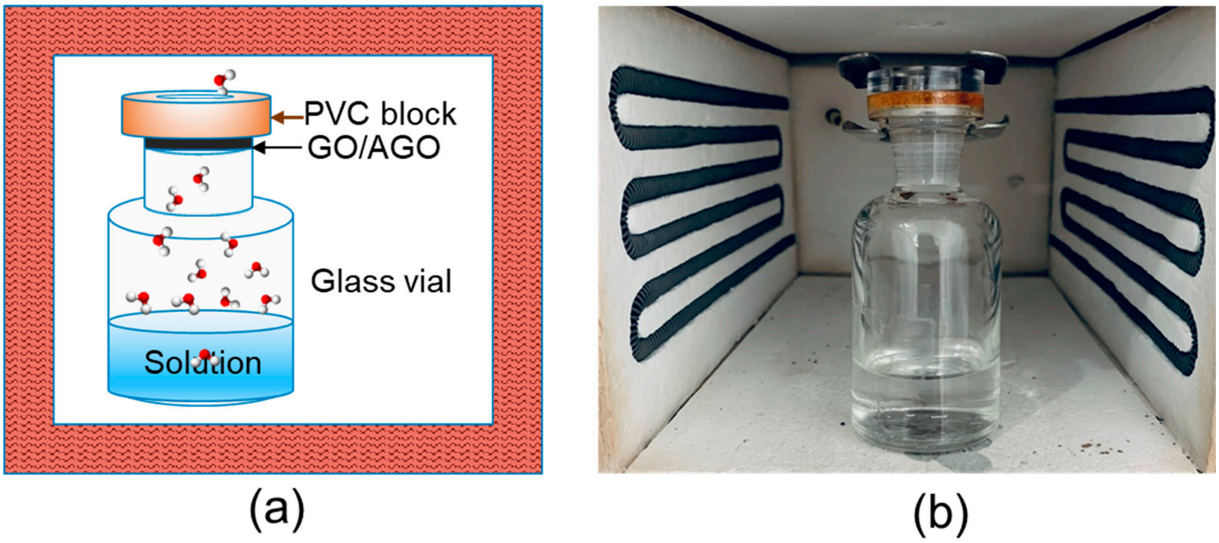

Figure S1. Schematic (a) and camera picture (b) of the permeability experiment set up.

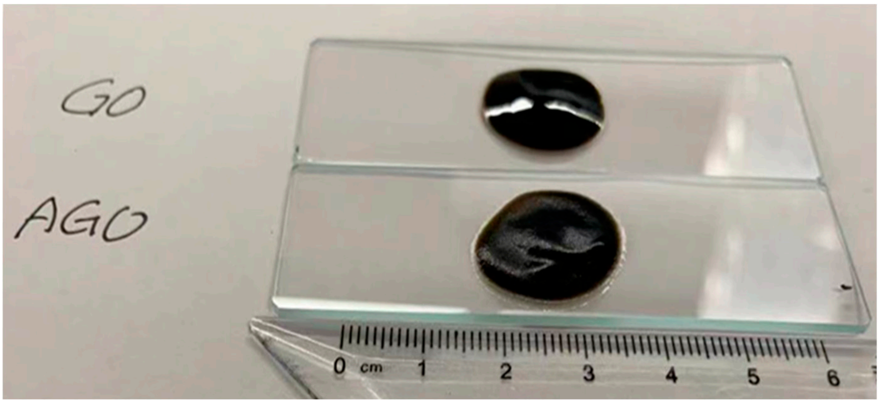

Figure S2. The AGO solution has a “muddy” look when compared with the GO solution.

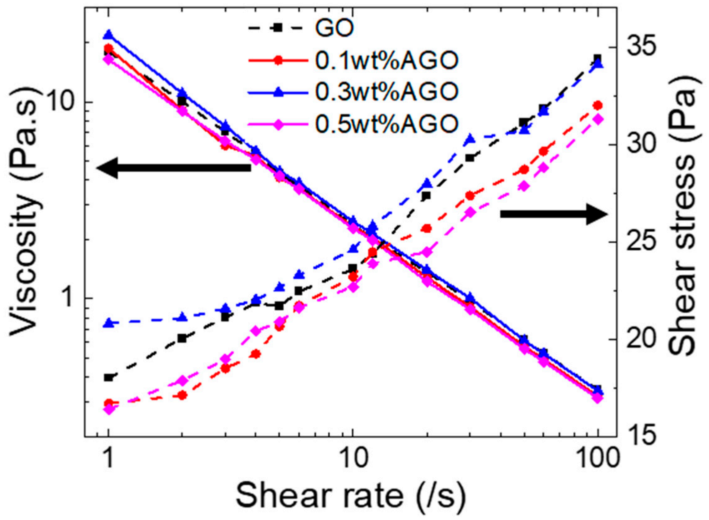

Figure S3. Viscosity and shear stress at different shear rates of GO solutions with Al concentration from 0~0.5wt%.

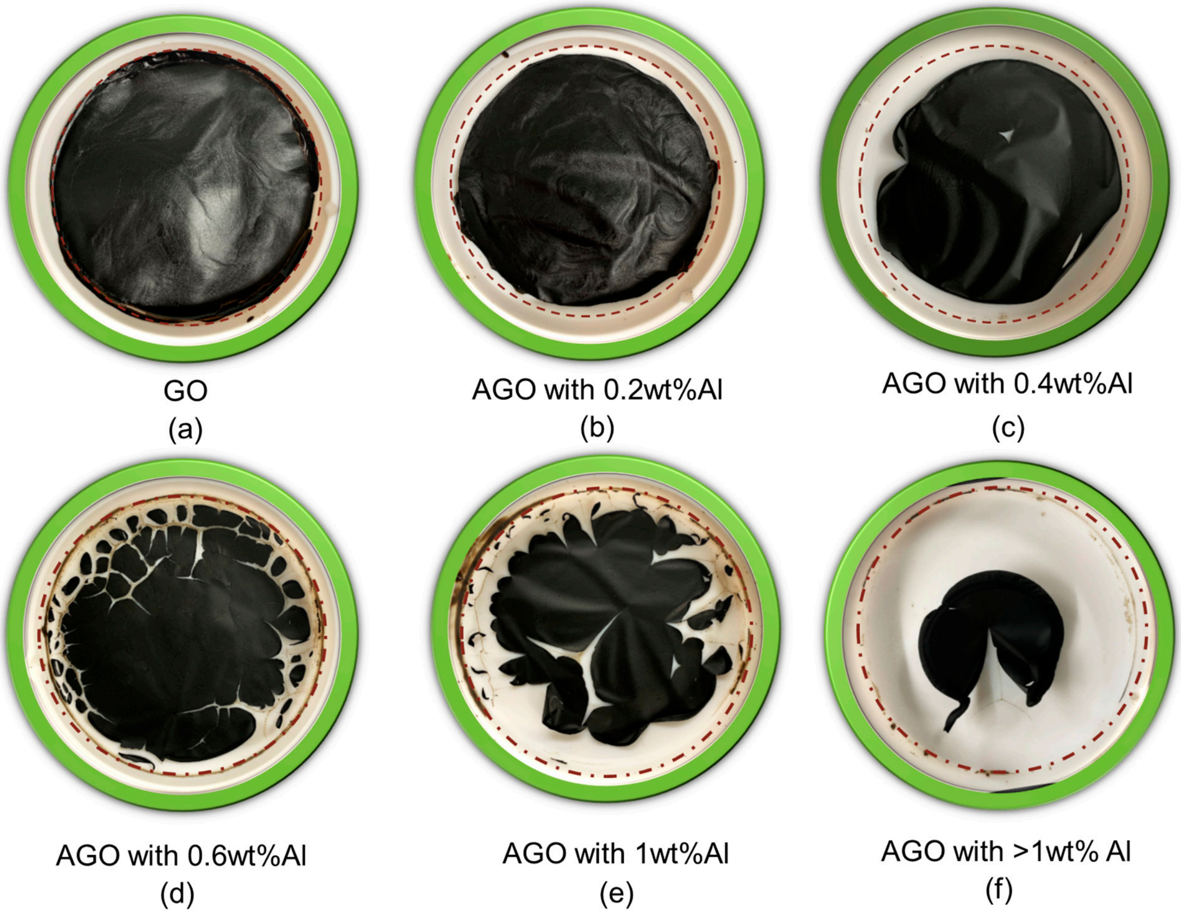

Figure S4. Digital camera image of as-fabricated GO and AGO membrane in the Teflon evaporation dish. (Dashed red circles indicate the inner circle of the evaporation dish, with a diameter of 12cm) (a) unmodified GO membrane(diameter of 12cm) has the same size as the evaporation dish (b) AGO membrane contains 0.2 wt.% Al(diameter of 11.7cm) is slightly smaller than the evaporation dish; (c) AGO membrane contains 0.4 wt.% Al (diameter of 11.1cm) shows both shrinking and cracking; more shrinking and crack can be observed on the AGO membrane contains (d) 0.6 wt.% Al; (e) 1 wt.% Al; and (f) more than 1wt. % Al.

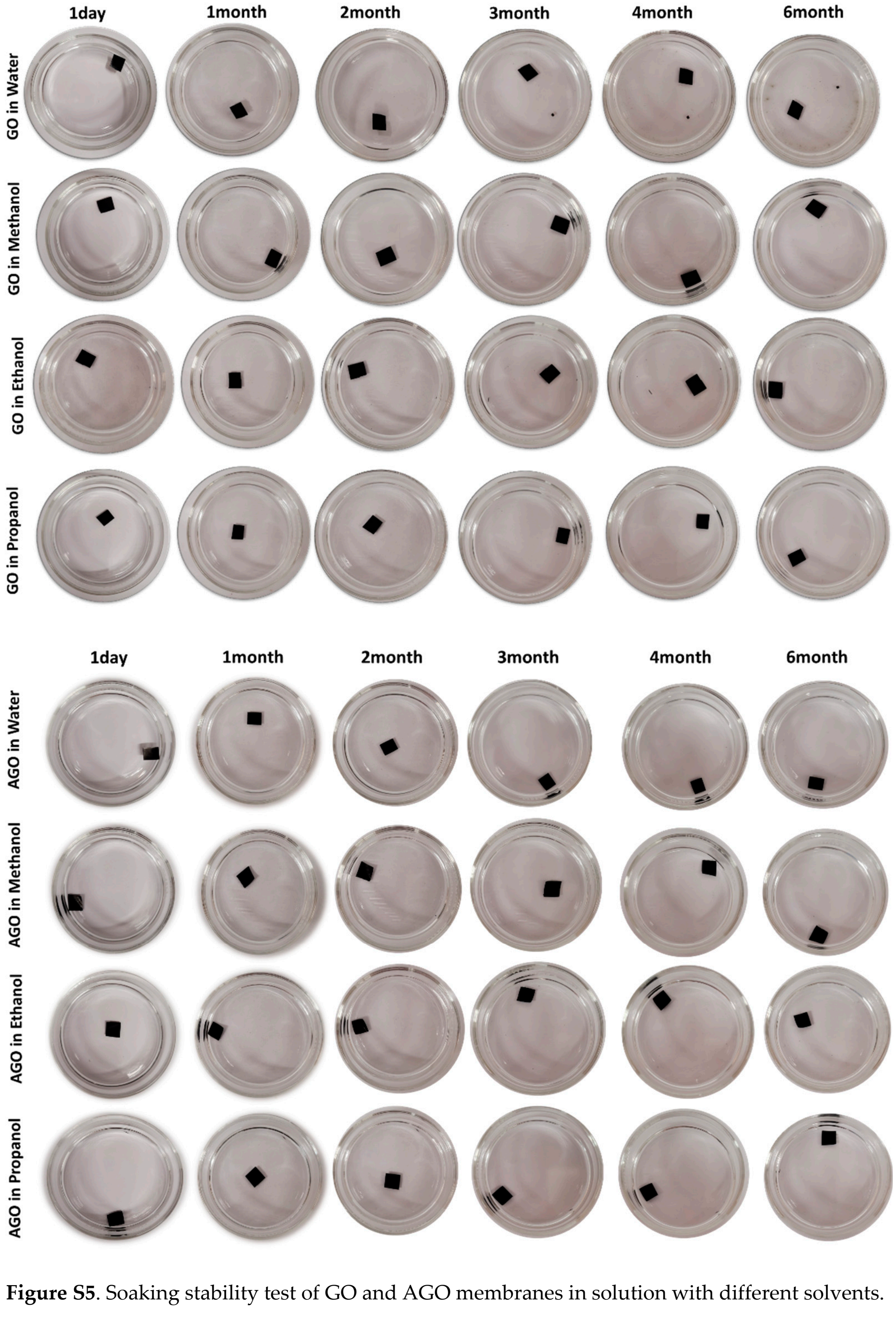

Figure S5. Soaking stability test of GO and AGO membranes in solution with different solvents.

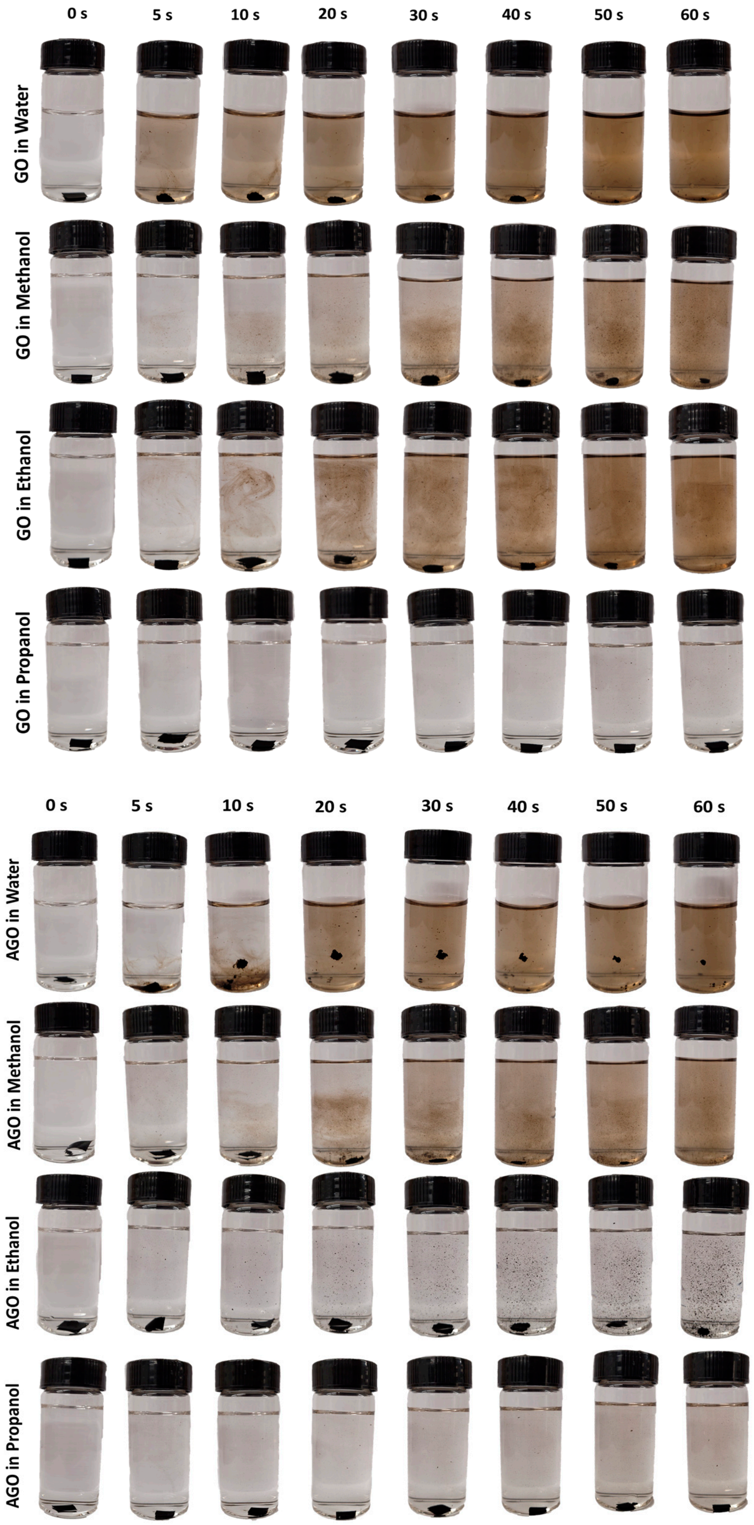

Figure S6. Sonication stability test of GO and AGO membranes in solution with different solvents.

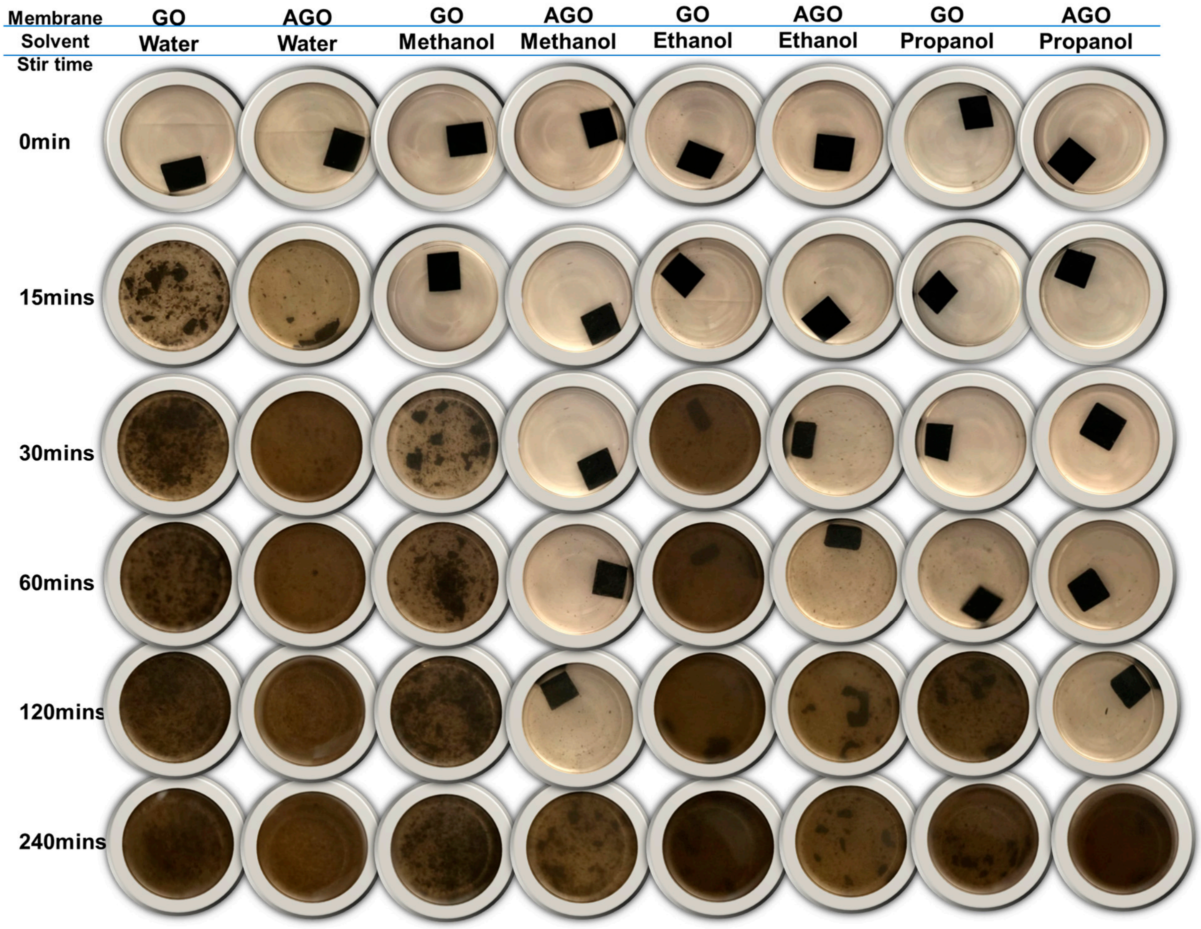

Figure S7. Stability test of GO and AGO membranes in different solvents with stirring rate of 200 rpm with large stir bar (12mm Length*5mm Diameter) touches the sample during stirring.

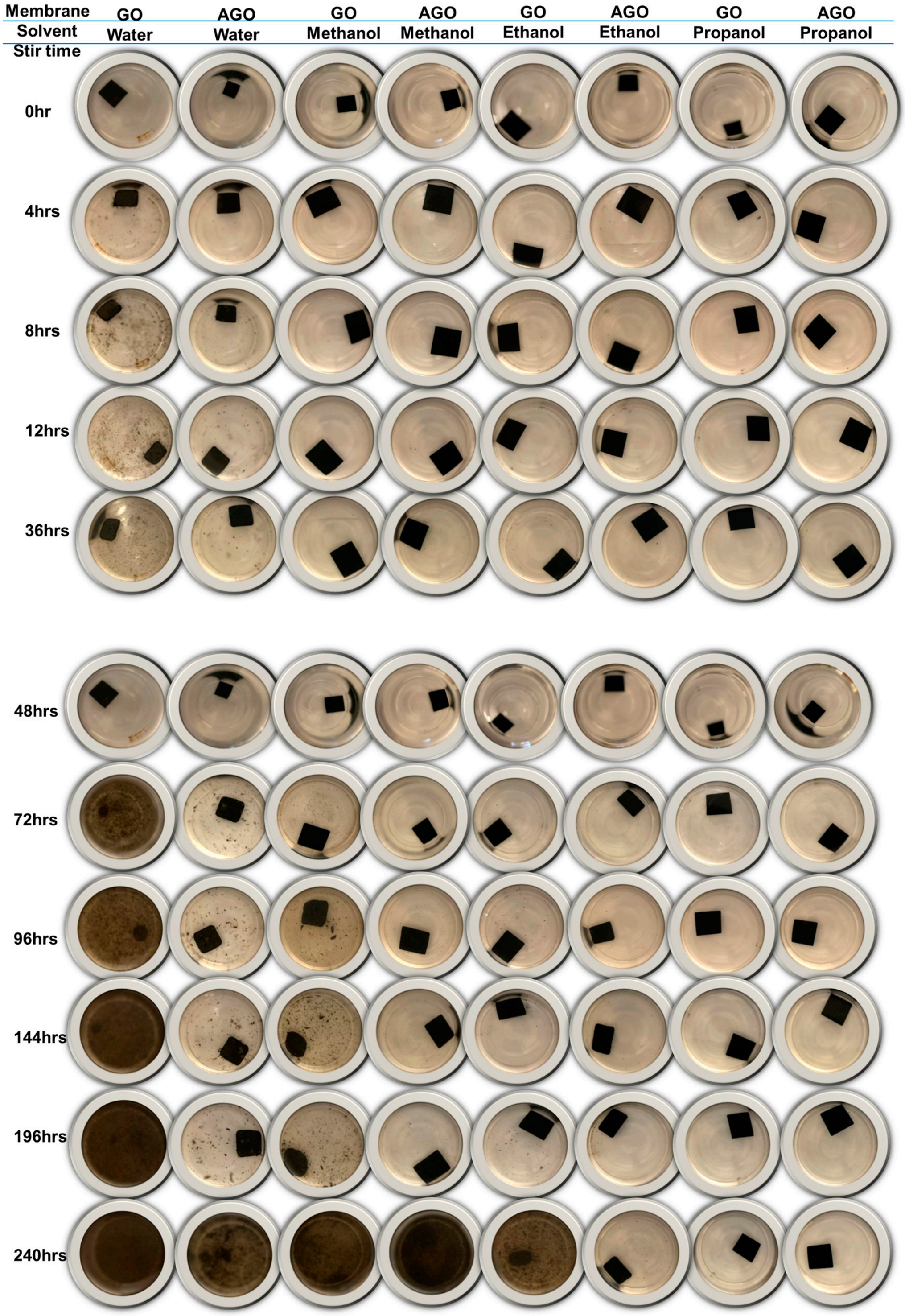

Figure S8. Stability test of GO and AGO membranes in different solvents with stirring rate of 200 rpm with small stir bar (10mm Length *3mm Diameter) bar not touch the sample during stirring.

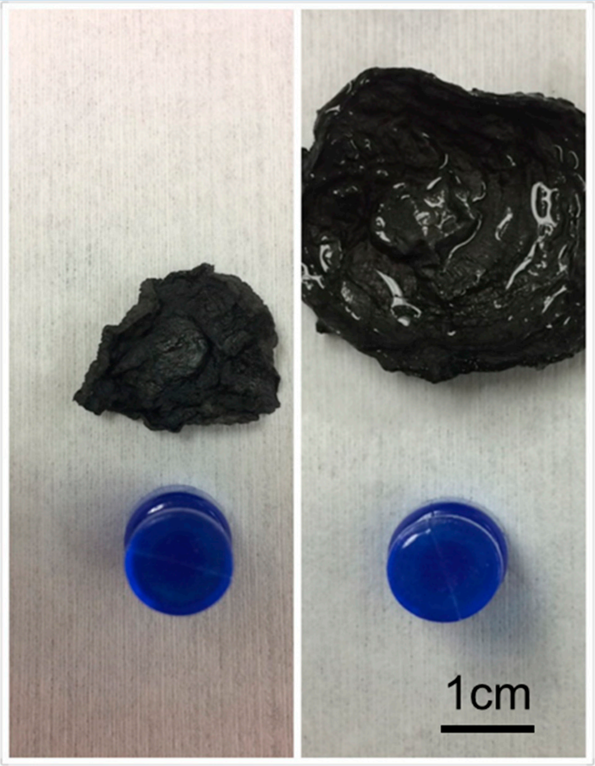

Figure S9. A highly wrinkled and contracted AGO membrane (left) swelled after soaking in water for 48 hours (right). The diameter of the AGO membrane increased 1.5 times, and the thickness increased 5 times.

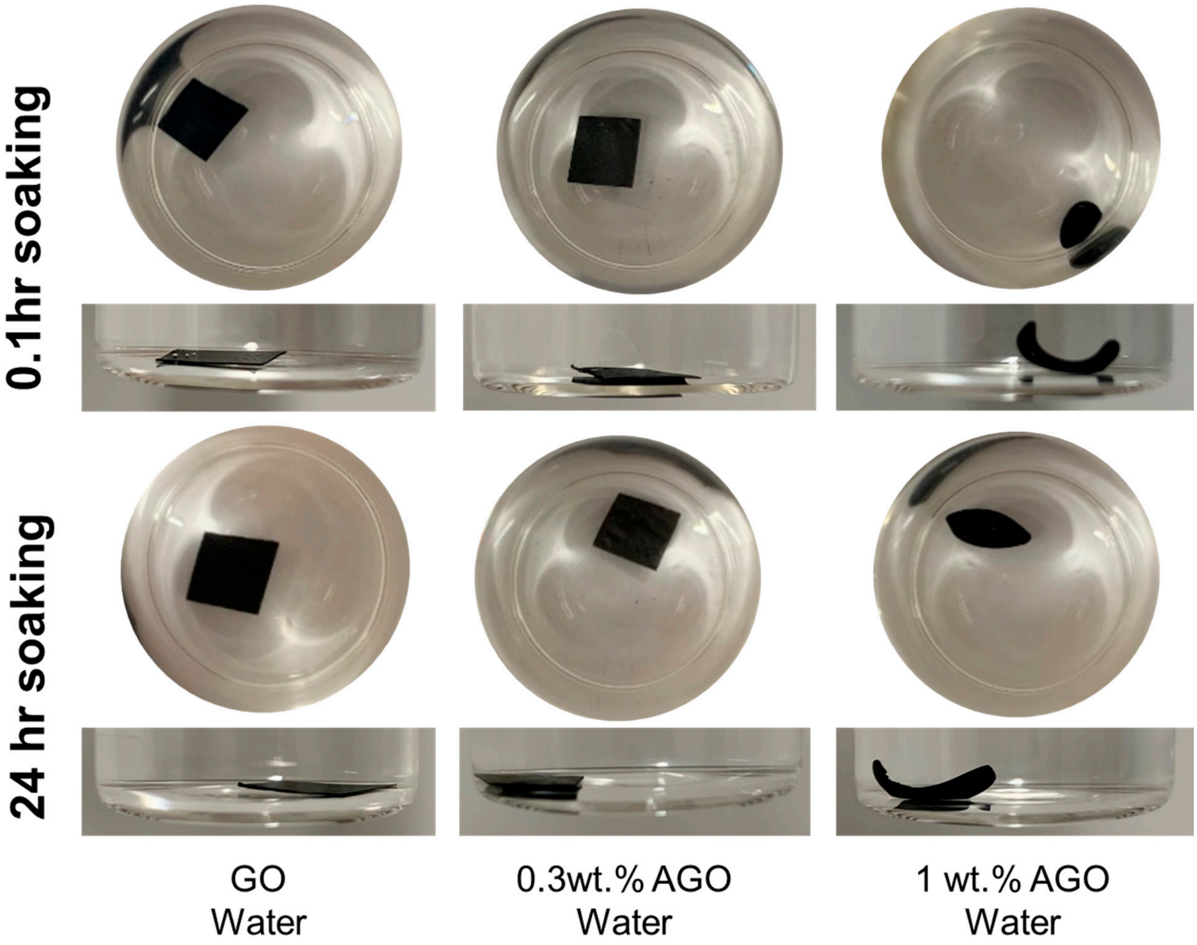

Figure S10. GO (left column) and AGO (middle and right columns) membrane soaking. The swelling cannot be visibly observed for the GO membrane and the AGO membrane with Al wt.%< 1wt.%

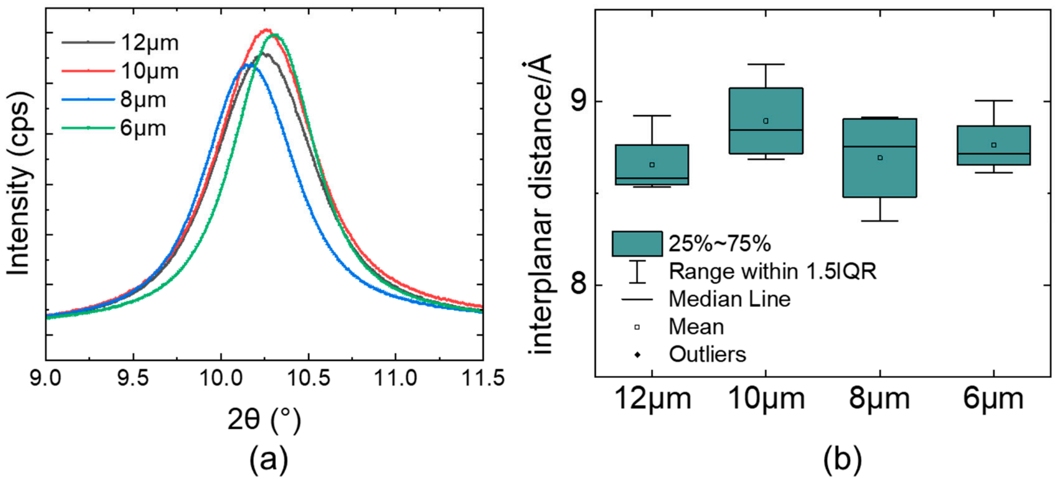

Figure S11. (a) XRD spectra of GO membrane with different thicknesses, and (b) distribution of interplanar distance derived from XRD spectra.

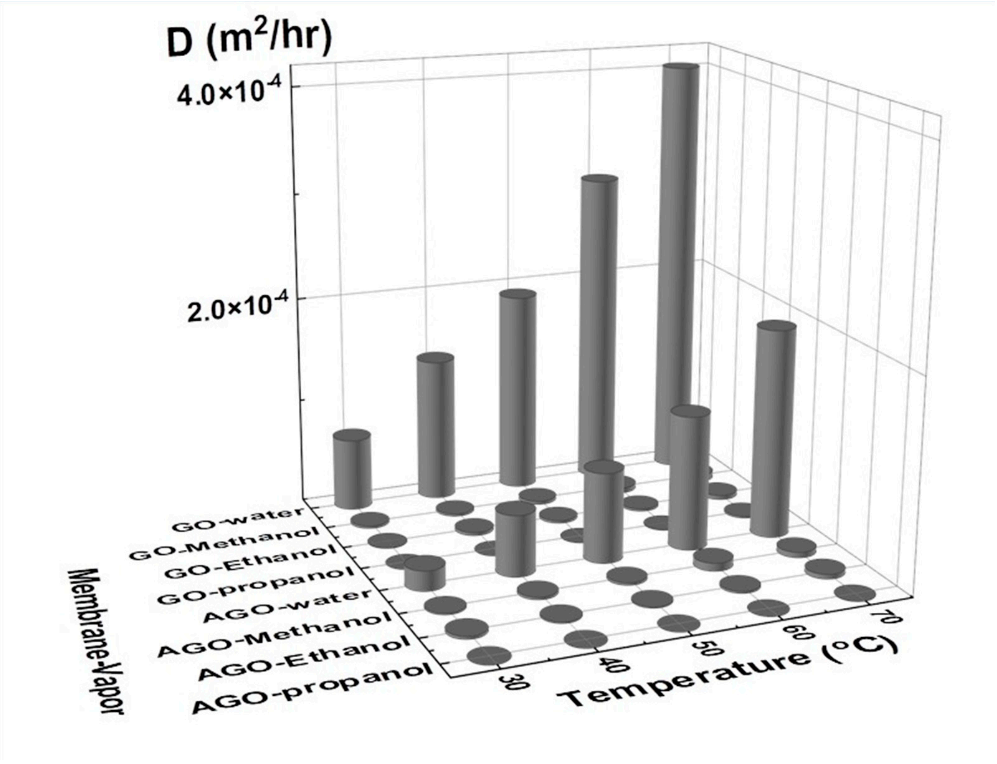

Figure S12. Permeability of GO and AGO membranes to water, methanol, ethanol, and propanol water at different temperatures.
